# The Role of Aging in Intracerebral Hemorrhage

**DOI:** 10.3390/brainsci14060613

**Published:** 2024-06-19

**Authors:** Baisong Huang, Anqi Chen, Yuanyuan Sun, Quanwei He

**Affiliations:** Department of Neurology, Union Hospital, Tongji Medical College, Huazhong University of Science and Technology, Wuhan 430022, China

**Keywords:** intracerebral hemorrhage (ICH), aging, senescence, hypertension, cerebral amyloid angiopathy (CAA), inflammation

## Abstract

Intracerebral hemorrhage (ICH) is the cerebrovascular disease with the highest disability and mortality rates, causing severe damage to the health of patients and imposing a significant socioeconomic burden. Aging stands as a foremost risk factor for ICH, with a significant escalation in ICH incidence within the elderly demographic, highlighting a close association between ICH and aging. In recent years, with the acceleration of the “aging society” trend, exploring the intricate relationship between aging and ICH has become increasingly urgent and worthy of in-depth attention. We have summarized the characteristics of ICH in the elderly, reviewing how aging influences the onset and development of ICH by examining its etiology and the mechanisms of damage via ICH. Additionally, we explored the potential impacts of ICH on accelerated aging, including its effects on cognitive abilities, quality of life, and lifespan. This review aims to reveal the connection between aging and ICH, providing new ideas and insights for future ICH research.

## 1. Introduction

Intracerebral hemorrhage (ICH) is characterized by the release of blood from ruptured or damaged cerebral blood vessels into the space within the brain tissue. This often leads to damage to the surrounding brain tissue, affecting neurological function and the normal functioning of the body. Although ICH accounts for only 10–15% of all strokes [1], its mortality rate can reach an alarming 30–40% [2]. Currently, the overall incidence rate of ICH is 24.6 cases per 100,000 people. There are significant differences in the incidence of ICH among different racial groups. In the United States, the risk of ICH among Black and Hispanic populations is approximately 1.6 times higher than that among White populations [3]. The incidence of ICH in Asian populations is nearly twice that in other ethnic groups, such as Black, Indian, Hispanic, Maori, and White populations [4]. Based on the mechanisms and etiology of ICH, it can be classified into two categories: primary and secondary ICH. Primary ICH is the most common type and occurs when blood vessels within the brain tissue rupture without any specific underlying pathology, leading to the leakage of blood into the brain tissue, forming a hematoma. Hypertension, cerebral amyloid angiopathy (CAA), and coagulopathies (the use of anticoagulant and antiplatelet drugs) are the primary causes of primary ICH. Secondary ICH can be caused by vascular malformations (cavernous angioma disease, Moyamoya disease), arteriovenous malformations, aneurysms, tumors, and hemorrhagic transformation of ischemic stroke or venous thrombosis [5].This article will focus solely on primary ICH.

Following an ICH event, brain tissue sustains various types of damage, which can be categorized as primary and secondary injuries. Primary injury is due to the direct mechanical damage caused by the hematoma’s compression on the brain, leading to increased intracranial pressure and potentially causing brain herniation [6]. Secondary injury can be broadly classified into two categories: the direct toxic effects of blood components and their metabolic byproducts and secondary excessive immune-inflammatory responses. Blood components and their metabolic byproducts can disrupt the blood–brain barrier (BBB), releasing substances such as hemoglobin, heme, heme oxygenase-1 (HO-1), and free iron, which contribute to brain tissue damage through oxidative stress and inflammatory reactions. Elevated coagulation factor levels in the blood can directly damage the BBB, leading to vasogenic edema. Simultaneously, blood, acting as a foreign substance, activates inflammatory cells like microglia, triggering an excessive inflammatory response. Inflammatory factors, such as IL-1 and IL-6, are released in large quantities, causing peripheral blood monocytes and other inflammatory cells to gather around the hematoma, resulting in neuroexcitotoxicity and neuronal cell death [7]. Although the primary and secondary injury pathways in ICH have distinct pathophysiological mechanisms, they interact and collectively contribute to brain tissue damage. 

Aging is a major risk factor for ICH, and as individuals grow older, the incidence of ICH increases [8]. However, most experimental studies on ICH have used young mice as animal models, and the impact of aging on ICH has not received sufficient attention. The prolongation of the human lifespan has brought about the burden of age-related diseases, including ICH. Moreover, with the declining global birth rates and the accelerating pace of population aging, ICH is becoming an increasingly significant challenge in the field of healthcare. Aging is also considered a significant risk factor for ischemic stroke (IS). With population growth and aging, the incidence, prevalence, and mortality rates of IS and ICH have markedly increased [9]. Aging can affect the occurrence and progression of ICH through the deterioration of vascular health and related comorbidities (such as hypertension, atherosclerosis, hypercholesterolemia, atrial fibrillation, and diabetes), and it similarly influences the occurrence and progression of IS. Therefore, studying the role of aging in ICH can also provide insights and guidance for IS research. We summarize the characteristics of ICH in elderly patients, reviewing the causative factors and the injury mechanisms of ICH to elucidate how aging influences the onset and progression of ICH. The paper concludes by exploring the potential impact of ICH on accelerating the aging process. It offers new research perspectives for ICH, aiding in the prevention and treatment of the damages caused by aging on ICH.

## 2. Characteristics of ICH in Geriatric Patients

### 2.1. High Incidence and Mortality Rates

**Figure 1:**
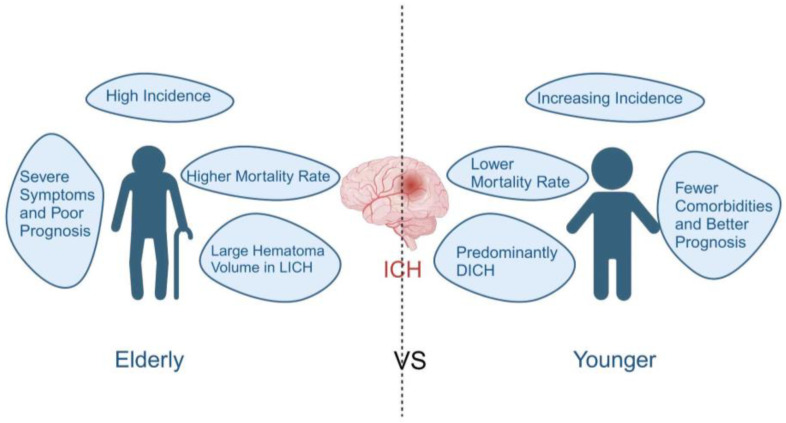
Characteristics of ICH in geriatric and young patients. Compared to young ICH patients, elderly ICH patients have a higher incidence and mortality rate, more severe clinical symptoms, and a worse prognosis [10,11]. Larger LICH hematomas are often found in elderly ICH patients [15]. Young ICH patients almost exclusively suffer from DICH caused by hypertension, and the onset age of ICH is becoming increasingly younger [14], which requires significant attention. Created with BioRender.com.

The risk of ICH is significantly higher in the elderly compared to younger individuals. An analysis of 52 studies from 28 countries, involving a total of 23,933,708 people, indicated that the incidence of ICH increases significantly with age. The incidence rate is 2.37 cases per 100,000 people for those under 45 years of age, 55 cases per 100,000 for those aged 55–64, 111.3 cases per 100,000 for those aged 65–74, 166 cases per 100,000 for those aged 75–84, and 95.4 cases per 100,000 for those aged 85 and above [10] (Figure 1). Research also shows that ICH patients aged over 80 have a 15.9% higher in-hospital mortality rate compared to those under 80 [11]. Of course, the incidence of ICH is closely related to ethnicity, region, and socioeconomic conditions. Globally, Asians have the highest proportion of non-traumatic ICH cases compared to most other ethnic groups [12], and low-income countries have an ICH incidence more than twice that of high-income countries [13]. In recent years, there has been a trend toward younger individuals and middle-aged people experiencing an increasing incidence of ICH. Studies indicate that the age-standardized incidence rates (ASIR) for individuals under the age of 65 increased significantly by 8.3% from 2005 to 2019 [14]. This may be attributed to the rising prevalence of hypertension in younger and middle-aged individuals who are unaware of their condition and, as a result, do not seek treatment for high blood pressure. However, ICH in younger and middle-aged individuals poses the risk of a longer-lasting disability, increased healthcare costs, and potential workforce losses for society. In summary, the incidence of ICH increases with age, and the proportion of ICH cases in middle-aged and younger individuals is also on the rise. Faced with the global trend of an aging population, it is imperative to pay sufficient attention to ICH.

The increasing probability of ICH with age is often due to the fact that as individuals age, they are more predisposed to the development of multiple chronic conditions, including hypertension and diabetes and coronary heart disease, which inherently increase the risk of ICH. Recent research has pointed out that chronic kidney disease (CKD) is independently associated with a high risk of ICH, and this connection is not influenced by ethnicity or race [16]. In addition, the medication used to treat these diseases can also affect the risk of ICH occurrence and the treatment of ICH. For instance, anticoagulants and antiplatelet drugs used for cardiovascular disease treatment are known risk factors for ICH.

A prospective study conducted in China has demonstrated a correlation between prior antithrombotic therapy and an elevated mortality risk in patients with ICH. This association is particularly strong among elderly patients over the age of 65 who had prior oral anticoagulant therapy [17]. Additionally, previous direct oral anticoagulant (DOAC) therapy is associated with increased in-hospital death and post-discharge death following ICH when compared to patients not using anticoagulants [18,19].

### 2.2. The Hematoma Volume and Bleeding Location Are Different from Those in Younger Individuals

The retrospective analysis of ICH patients revealed a significant increase in hematoma volume among elderly ICH patients [20]. The larger the hematoma volume, the greater the mechanical damage to the surrounding brain tissue. Hematomas may also exert pressure on adjacent brain blood vessels, leading to cerebral ischemia, hypoxia, and even death. Large subcortical hematomas are common in ICH patients aged over 80, and their prognosis is relatively poorer [21]. Research has found that lobar ICH (LICH) has a larger hematoma volume than deep ICH (DICH), and the hematoma volume of LICH increases with the patient’s age, while deep-seated hemorrhages are unaffected by age [15]. This may be attributed to cerebral parenchymal degeneration during the aging process, reducing the structural integrity of brain tissue and making hematoma growth easier and less constrained. Overall, a larger hematoma volume may impact brain tissue function and survival at multiple levels, thereby increasing the mortality rate of ICH patients. Therefore, in the treatment and management of ICH, the early and effective reduction in hematoma volume may be a key factor in reducing the mortality rate and improving patient prognosis.

The brain structure of elderly individuals can vary due to age- and disease-related changes, and different locations of ICH bleeding may result in different symptoms and clinical manifestations. Research indicates that in elderly ICH patients, bleeding often occurs in the cerebral lobes, whereas in younger individuals, ICH bleeding is more common in deep-seated areas [21]. This difference is primarily due to the prevalence of hypertensive ICH in younger individuals, which often occurs in the deep basal ganglia region of the brain. In contrast, CAA is highly common in the elderly but rare in younger individuals, and CAA-related bleeding is known to occur in the lobes at an early stage [22]. Further research on the location of ICH bleeding has revealed that it is not only associated with age but may also be related to the burden of small vessel disease (SVD). This research suggests that younger patients are more likely to experience striatal hemorrhages, while elderly individuals and those with a low SVD burden tend to have more LICH. On the other hand, elderly patients with a high SVD burden often experience thalamic hemorrhages [23].

Research on the location of ICH bleeding remains insufficient, and further studies are necessary as they can provide a better understanding of ICH and contribute to its treatment.

### 2.3. More Severe Clinical Symptoms and Worse Prognosis

The fact that elderly individuals with ICH exhibit more severe clinical symptoms and experience a worse prognosis is undeniable. This can be attributed to the more significant damage caused by larger hematoma volumes in the elderly, the use of anticoagulant medications, and their diminished physiological reserve (the body’s capacity to endure and overcome injuries).

A comparative analysis of ICH patients across different age groups revealed that the National Institute of Health stroke scale (NIHSS) score increases with age. Additionally, in the elderly, hematoma volume is independently associated with three-month mortality rates, a correlation not found in younger patients [24]. A multicenter clinical study found that the use of antithrombotic drugs in hypertensive ICH patients increases with age. Moreover, older ICH patients present with more severe symptoms upon admission and experience worse functional outcomes. This may be linked to the increasing rates of antithrombotic therapy with age [25].

Aging is not only reflected in changes in the nervous system but also manifests in other systems. Other age-related comorbidities are key factors that complicate the treatment and lead to poorer outcomes in elderly patients with ICH. Gaist et al. compared 8991 previous ICH patients from Denmark with an average age of 70.7 years to 359,185 age- and sex-matched control group members, finding that the main comorbidities in ICH patients included hypertension (67.7% vs. 51.5%), atrial fibrillation (14.7% vs. 7.6%), previous IS (13.6% vs. 3.2%), diabetes (11.7% vs. 10.4%), and chronic kidney failure (2.1% vs. 1.5%) [26]. Furthermore, Vanent et al. found, through a multicenter study in the United States and a population-based study in the United Kingdom, that CKD is consistently associated with a higher risk of ICH in populations with an average age over 56 years, and this association is causal [16].

In addition to the aforementioned main comorbidities, Kim et al. compared 25,810 elderly ICH patients (>45 years) with 1929 young ICH patients (≤45 years) and found that elevated cholesterol (39.86% vs. 15.4%) was also one of the common comorbidities in elderly ICH patients [27]. Moreover, Zhang et al.’s study on 248 ICH patients aged 70 years and above found that certain pulmonary diseases (such as chronic obstructive pulmonary disease, chronic bronchitis, and pulmonary fibrosis) and cerebrovascular diseases (such as previous IS and transient ischemic attack) were common comorbidities in ICH, significantly impacting patient prognosis and treatment strategies. The Charlson Comorbidity Index (CCI) is used to quantify the impact of these comorbidities and is associated with short-term prognosis and hospital mortality in patients [28].

The common characteristic of these comorbidities in the elderly is chronic damage and changes to the vasculature, making the vessel walls fragile and increasing the likelihood of ICH. These comorbidities also complicate the treatment of elderly ICH patients and lead to poorer prognoses.

## 3. Aging and Its Impact on ICH

### 3.1. Hypertension

**Figure 2:**
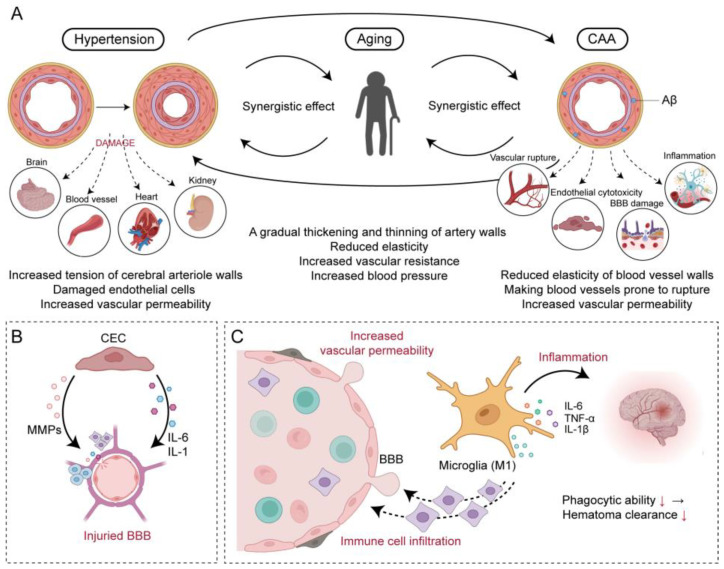
Aging affects the occurrence and development of ICH. (**A**) demonstrates the synergistic effects between hypertension, CAA, and aging. The occurrence of hypertension leads to thickening and hardening of cerebral arteriolar walls, lumen constriction, and reduced vascular wall elasticity, which also impacts other organs in the body. Following CAA, Aβ deposition in the vascular media causes basement membrane thickening, lumen narrowing, and decreased vascular wall elasticity, inducing inflammation and endothelial cell toxicity, thereby leading to BBB damage. (**B**) shows that aging brain endothelial cells release pro-inflammatory substances such as MMPs, IL-1, and IL-6. These substances disrupt the connections between CECs, exacerbating BBB damage. (**C**) illustrates that aging microglia tend to exhibit a pro-inflammatory M1 phenotype, secreting inflammatory factors, inducing neuroinflammation, and increasing the infiltration of exogenous immune cells into the BBB, further damaging the BBB. Additionally, aging microglia have reduced phagocytic capacity, resulting in decreased clearance of hematomas post-ICH, which exacerbates brain damage caused by ICH. Created with BioRender.com.

Hypertension is a widely acknowledged cause of ICH and, concurrently, the most significant modifiable risk factor for ICH. Research indicates that untreated high blood pressure can increase the risk by 2 to 4 times, including ICH and other stroke risks. In patients under 70 years of age, chronic hypertension is the primary cause of ICH [29]. High blood pressure leads to ICH primarily through the mechanism of hypertensive arteriopathy. Significantly elevated blood pressure within the brain increases tension in the walls of small cerebral arteries, damages endothelial cells, and increases vascular permeability. Plasma proteins infiltrate the vessel wall and clot beneath the endothelial cells, forming an amorphous, eosinophilic, hyaline material known as fibrinoid necrosis. The accumulation of these changes can result in thickening and stiffening of the walls of small cerebral arteries and constriction of their lumens, ultimately reducing vascular wall elasticity and increasing intraluminal pressure, leading to rupture and bleeding [30] (Figure 2).

Recent animal experimental studies have shown a synergistic effect between aging and hypertension, exacerbating the occurrence of ICH. The mechanism may involve age-related oxidative stress in cerebral blood vessels induced by hypertension and the oxidative/redox-sensitive activation of matrix metalloproteinases (MMPs) [31]. With advancing age, the impact of aging on the development and progression of hypertension becomes increasingly evident. Aging first results in irreversible alterations to the structure and function of blood vessels, characterized by gradual arterial wall thickening, thinning, decreased elasticity, and elevated vascular resistance, promoting increased blood pressure. Additionally, aging leads to reduced fluid regulatory function within the body. Between the ages of 25 and 85, approximately 40% of renal units develop sclerosis. Moreover, renal blood flow decreases by half, glomerular filtration rates decrease to around 45%, and renal sodium excretion function declines, leading to an increase in blood volume and further exacerbating the development of hypertension [32]. Aging may also lead to increased sympathetic nervous system excitation and reduced parasympathetic nervous activity, affecting the stability of the cardiovascular system, thereby contributing to hypertension.

Conversely, hypertension also accelerates the aging process. High blood pressure can cause brain damage, including vascular injury and cerebral ischemia, further accelerating brain tissue aging and potentially leading to cognitive decline. Long-term hypertension imposes an increasingly significant strain on the cardiac and circulatory systems, accelerating the senescence of the cardiovascular apparatus and heightening the likelihood of cardiovascular pathologies. Furthermore, hypertension significantly damages the kidneys, leading to a decline in glomerular filtration rates and renal function, further accelerating the aging process.

These interconnected processes demonstrate the close relationship between hypertension and aging, exerting profound effects on the functionality and overall health of multiple organ systems. Therefore, the prevention and control of hypertension are critically important for delaying the aging process and preventing the occurrence of ICH. According to the World Health Organization’s “Global Hypertension Report”, as of 2019, there were already 1.3 billion global hypertension patients. However, nearly half of these patients were unaware of their condition, and approximately four-fifths did not receive adequate treatment [33]. Moreover, older adults account for a majority of the incidence and mortality related to hypertension, largely due to the significantly higher prevalence of the condition within this population. Data show that 70% of adults aged 60 and over have hypertension, compared to only 32% of adults aged 40 to 59 [34]. As the global population continues to age, the harm caused by hypertension and ICH will become more severe. It is crucial to give this issue the attention it deserves and actively manage hypertension.

### 3.2. CAA

CAA refers to the deposition of beta-amyloid (Aβ) in the middle and outer membranes of the small- and medium-sized arteries in the cerebral cortex and medulla. The vast majority of CAA cases are sporadic, mainly manifesting as ICH and cognitive disorders in the elderly. CAA is quite common in old age. Nearly a quarter of autopsy brains in a general population with an average age of 84.9 years were found to have moderate to severe CAA. Its prevalence is 48% among Alzheimer’s Disease (AD) patients, 19% to 24% in ICH patients, and as high as 50% to 57% in patients with LICH [35]. CAA-related LICH is characterized by a high recurrence rate, about 7.4% [36]. The deposition of Aβ damages the middle and outer membranes of blood vessels, leading to thickening of the basement membrane, narrowing of the vascular lumen, and loss of smooth muscle cells. This results in fibrinoid necrosis and decreased elasticity of the vessel wall, making the vessels prone to rupture and thus causing ICH. The deposition of Aβ not only affects the hemodynamics of cerebral blood vessels but also has a toxic effect on the brain and surrounding endothelial cells [37], causing endothelial dysfunction, oxidative stress, and various inflammatory changes.

Aging constitutes a primary risk factor for CAA. Research in non-demented populations has demonstrated an age-related increase in Aβ deposition, which is closely correlated with the progression of arterial stiffness [38]. This suggests an increase in the fragility of blood vessel walls and concurrent changes in the structure and function of cerebral blood vessels. The loss of normal cells in the vessel wall impairs vascular reactivity, potentially reducing the brain’s ability to clear Aβ and contributing to the development of CAA [39,40]. Aβ deposition may also impair the function of the BBB. Studies have shown that the breakdown products of amyloid precursor protein damage pericytes and increase vascular permeability [41,42]. BBB impairment increases the likelihood of toxins and inflammatory factors entering the brain tissue, triggering an inflammatory response in the cerebral vessel wall, further damaging it and promoting the progression of CAA. In terms of inflammation and innate immune response, the deposition of Aβ is considered a driver of neuroinflammation, with clusters of activated microglia found around Aβ-laden capillaries [43]. Following the inflammatory response, activated microglia induce the production of reactive oxygen species (ROS) through NADPH oxidase (NOX). The increase in ROS leads to the loss of endothelial cell–cell interactions and the disruption of BBB integrity via tight junctions (TJs). Perivascular macrophages that contribute to ROS production have been found in the perivascular spaces of AD mouse models (sites of Aβ transport and accumulation in the brain). These macrophages are the main source of ROS production in the brain vasculature following Aβ deposition, altering neurovascular function through ROS generation, oxidative stress induction, and their receptors CD36 and Nox2 [44].

Endothelial cells and smooth muscle cells also play crucial roles in CAA-related inflammatory responses. The deposition of Aβ not only triggers structural and functional changes in these cells but also promotes inflammation and neurovascular unit dysfunction by activating cell death receptors and secreting inflammatory mediators [45].

In terms of adaptive immune response, in AD mouse models, although T cells have been observed to infiltrate brain regions with Aβ deposition, there are obstacles to antigen presentation and T-cell activation within the brain, preventing T cells from providing the protective effects of a true adaptive immune response [46]. However, clonally expanded CD8 T cells have been found in the cerebrospinal fluid of AD patients, suggesting that T cells may be present on the surface of meningeal vessels prone to CAA [47]. Further research is needed to understand the adaptive immune response to Aβ deposition.

CAA is closely linked to hypertension. The deposition of Aβ not only increases the fragility of cerebral arterial walls but also impairs the reactivity of peripheral arteries [48], ultimately leading to vascular aging and hypertension. Hypertension, in turn, enhances the processing of amyloid precursor proteins into amyloid proteins [49]. This suggests that CAA and hypertension may synergistically accelerate the aging of cerebral blood vessels.

### 3.3. Aging Affect Injury Mechanisms of ICH

#### 3.3.1. Vascular Wall Genetics Associated with ICH

We have observed that both hypertension-induced DICH and LICH primarily caused by CAA are crucially linked to damage to the cerebral vascular wall. This damage is either the direct cause of ICH or it plays a significant role in the development and progression of ICH. Some genetic variants might be the culprits, as their continual contribution to the damage of cerebral vascular walls, with advancing age, accelerates the onset and progression of ICH.

Apolipoprotein E (APOE) plays a crucial role in lipid transport and metabolism, mainly synthesized in the liver and by astrocytes in the brain. The APOE gene is located on chromosome 19q13.32 and has three common alleles (ε2, ε3, and ε4). APOE, through its ε2 and ε4 alleles, increases the deposition of Aβ in the cerebral vascular wall, thus leading to CAA-related vascular pathology and an increased risk of ICH [50]. Studies by Tzourio and others have shown that carrying ε2 or ε4 alleles increases the risk of both DICH and LICH, and this risk is race-dependent. For Asians, the risk of DICH is doubled, while for Europeans, the risk of LICH is tripled for carriers of ε2 or ε4 [51]. Research by Biffi and others on a European cohort (2189 ICH cases and 4041 controls) identified both ε2 and ε4 as risk factors for LICH, with ε4 also being associated with DICH [52]. A meta-analysis incorporating eleven case–control studies indicated that the frequency of the APOE ε4 allele is significantly higher in both Asian and Caucasian ICH cases, whereas no significant relationship was found between the APOE ε2 allele and ICH risk [53]. Similarly, research by Nie and others considers both ε2 and ε4 APOE alleles as risk factors for ICH in Caucasians, but not in Asians [54].

In summary, multiple studies indicate that the APOE ε4 allele is a risk factor for ICH, increasing the risk of ICH, whereas the association of the APOE ε2 allele with increased ICH risk remains controversial. Whether carrying ε2 or ε4 APOE alleles genuinely increases ICH risk across different races still requires further research. APOE’s role in increasing Aβ deposition in the cerebral vascular wall and promoting cellular senescence was also highlighted by Zhao and others, who found that APOE accumulation is a driver of cellular aging in mesenchymal progenitor cells (MPCs) among the elderly, whereas the absence of APOE enhances these cells’ resistance to aging [55]. This suggests that APOE-mediated Aβ deposition and the promotion of endothelial cell aging may jointly contribute to vascular wall damage.

Angiotensin I Converting Enzyme (ACE) is an enzyme that plays a critical role in blood pressure regulation and fluid balance. ACE catalyzes the conversion of angiotensin I to angiotensin II, which causes vasoconstriction, thus increasing vascular resistance and blood pressure. Prolonged vasoconstriction can lead to damage and hardening of the vascular walls, thereby increasing the risk of ICH. The ACE gene is located on human chromosome 17q23.3 and exhibits various polymorphisms, with the insertion/deletion (I/D) polymorphism being the most extensively studied. Research by Kalita et al. has shown that the ACE DD genotype is significantly associated with hematoma occurrence in DICH [56]. The ACE DD genotype has a more defined impact on the occurrence of ICH in Asian and European populations compared to APOE. Studies by Kumar et al. found that the ACE gene I/D polymorphism is significantly associated with ICH risk, especially in Asians, where the DD genotype correlates with higher serum ACE levels, leading to vasoconstriction and endothelial dysfunction, thereby increasing the likelihood of ICH [57]. Additionally, a meta-analysis including 28 studies involving 2806 cases and 3612 controls indicated that the ACE gene I/D polymorphism’s DD homozygous genotype is associated with an increased risk of ICH in Asian populations and may have a synergistic effect with hypertension, while no such association was observed in Caucasian populations [58]. Similarly, research by Li et al. also suggests that the ACE I/D polymorphism affects the susceptibility of Asian individuals to ICH, but no association was found in Europeans [59].

Current studies indicate that the ACE gene I/D polymorphism is significantly associated with an increased risk of ICH in Asian populations, but no significant correlation has been found in Europeans. The ACE DD genotype is not only related to the occurrence of DICH but has also been linked to recurrent DICH. Research by Misra et al. has found that hypertensive patients with the ACE DD genotype or the D allele have a significantly higher risk of recurrent ICH [60]. Although the D allele of ACE is associated with the occurrence of ICH, individuals carrying the D allele are actually more likely to have longer lifespans (this can be considered a form of healthy aging). A meta-analysis including 1803 centenarians and 10,485 non-centenarians, as well as another meta-analysis involving 2043 individuals aged 85 and older versus 8820 younger controls, both confirmed a positive correlation between the D allele and longevity [61,62].

Collagen type IV is a major component of the vascular basement membrane, composed of type IV collagen α1 and α2 chains (COL4A1 and COL4A2). Mutations in COL4A1 and COL4A2 are increasingly recognized as causes of multisystem diseases, including ICH. The rs544012 AC and rs679505 AA genotypes of COL4A1 are independently associated with the risk of ICH in the Chinese Han population with hypertension, and the AA haplotype (rs3742207-rs11069830) in the COL4A1 gene may also be related to ICH risk among this group [63]. In another study, 1.94% (11/568) of Chinese ICH patients were found to have rare non-synonymous variants in COL4A1, with rs3742207 possibly indicating a poorer prognosis for ICH [64]. A meta-analysis involving 21,500 cases and 40,600 controls found that nine intronic single-nucleotide polymorphisms (SNPs) in COL4A2 are associated with an increased risk of symptomatic small vessel disease, especially DICH [65]. It is currently believed that mutations in the COL4A2 gene lead to the abnormal accumulation of COL4A1 and COL4A2 proteins in the endoplasmic reticulum, thereby affecting their normal secretion. These proteins, which should form part of the vascular basement membrane, when their secretion is obstructed, may cause structural defects in the basement membrane. Such structural defects weaken the stability and function of blood vessels, increasing vascular fragility, thereby promoting the occurrence and progression of ICH [66].

Matrix metalloproteinases (MMPs) and tissue inhibitors of metalloproteinases (TIMPs) are also focal points in the study of genetic variations affecting vascular wall integrity. MMPs are enzymes capable of degrading the extracellular matrix, with one crucial function being the regulation of the blood–brain barrier’s permeability, which is especially critical in the development of brain injuries and certain brain diseases. TIMPs are natural inhibitors of MMPs, responsible for balancing and regulating MMP activity, thus preventing excessive degradation of the extracellular matrix and maintaining the integrity and stability of tissue structures. An imbalance between MMPs and TIMPs can lead to the degradation of the ECM and vascular basement membranes, compromising the integrity of cerebral vascular walls and thereby promoting the occurrence of ICH. An imbalance between MMPs and TIMPs has also been observed in the formation of cerebral aneurysms, which may be due to ECM degradation leading to the progression and rupture of these aneurysms [67]. TIMP1 and TIMP2 are the main endogenous inhibitors of MMP9 and MMP2, respectively, encoded by the genes TIMP1 and TIMP2 [68]. Studies have shown that using MMP inhibitors in a mouse model of ICH can alleviate acute brain injuries, suggesting a potential therapeutic role for MMP inhibitors in treating ICH [69]. Variations in the promoters of MMP-2 and TIMP-2, as well as polymorphisms in MMP-9 and TIMP-1, have been found to be significantly associated with the risk of DICH, with notable age differences [70,71]. Research on the Chinese male population has identified the C allele of TIMP-1 rs2070584 as a risk factor for ICH [72]. In studies on Caucasian populations, the A allele of SNP–261G/A in the TIMP-2 gene has been significantly associated with an increased risk of ICH [73].

#### 3.3.2. Cerebrovascular Aging and BBB Damage

The BBB serves as an intermediary station between circulation and the central nervous system (CNS), composed of brain microvascular endothelial cells, astrocytes, basement membrane, and pericytes.

Aging induces structural modifications in blood vessels, impairs endothelial cell function, and disrupts the integrity of the BBB, a phenomenon confirmed in both humans and aging rodents [74,75,76]. The manifestations of aging-related BBB disruption in humans and animals involve reduced tight junction proteins [77], decreased pericytes [78], and pericyte-induced endothelial gene expression [79]. Aging can also inflict damage on the BBB through blood components. Research has revealed that aged donor red blood cells produce more nitric oxide (NO) under physiological shear stress compared to young donor red blood cells. NO, in turn, affects the expression of TJs, increasing the permeability of the BBB [80].

As people age, the aging of the human body not only affects the function of cerebral blood vessels, such as the reduced efficiency of glycolysis in cerebral microvessels [81], but also leads to changes in the integrity of cerebral blood vessels. Senescent cerebral endothelial cells (CECs) produce a senescence-associated secretory phenotype (SASP) [82], which not only secretes MMPs capable of degrading the extracellular matrix [83], but also releases pro-inflammatory substances such as IL-1 and IL-6. These substances disrupt the connections between CECs, thereby exacerbating cerebral vascular damage and facilitating the infiltration of exogenous immune cells and monocytes [84]. This process contributes to sustained low-grade chronic inflammation in cerebral tissues and vasculature.

Concurrently, senescent immune cells exhibit the increased secretion of ROS, stimulate the NF-kB signaling pathway in CEC [85], and promote fibrosis and collagen deposition. These changes further weaken the integrity of the cerebral blood vessels. Once the integrity of cerebral blood vessels is compromised, their ability to clear harmful molecules (such as Aβ) is diminished, exacerbating the development of CAA, thereby posing a threat to the function of neurons and cerebral vessels. Additionally, with aging, the MMPs secreted by senescent CECs, poor remodeling of the vascular wall, and the impaired expression of growth factors may lead to the distortion of cerebral blood vessels [83]. This can cause changes in hemodynamics, consequently increasing the risk of ICH.

It is noteworthy that in the elderly, especially those with neurodegenerative diseases such as AD and Parkinson’s Disease (PD), the disruption of the BBB is more pronounced [86]. In the brain tissue of individuals with AD, a subgroup of angiogenic endothelial cells has been observed, which exhibit increased expression of angiogenic growth factors and their receptors (EGFL7, FLT1, and VWF) and antigen-presenting mechanisms (B2M and HLA-E) [87]. This indicates an increasingly apparent contribution of CECs to the processes of angiogenesis and immune response within the context of AD and other analogous vascular pathologies. Concurrently, an augmentation in the population of CD8+ T effector memory CD45RA+ (TEMRA) cells is observed in patients diagnosed with Alzheimer’s Disease [47], reflecting changes in adaptive immune responses. In patients with vascular dementia, CECs have also been observed to generate pro-thrombotic mediators and cell adhesion molecules [88,89,90], enhancing the ability of immune cells to enter the central nervous system. This suggests that cerebrovascular damage affects the dynamics of immune cells in the brain, leading to an increased entry of cells involved in adaptive immune responses. In both animal models and humans, there is a noted elevation in the quantity of immune cells within the senescent brain, including dendritic cells, T cells, and B cells [91]. As the permeability of the BBB increases and its integrity is compromised, the elderly are more severely affected during ICH. Blood and its metabolic products can cause more severe damage to the fragile BBB, exacerbating inflammatory responses and making recovery prognosis more difficult.

#### 3.3.3. Age-Induced Immunosenescence and Increased Inflammation

The main reason for the difference in prognosis between young and elderly ICH patients may be due to changes in the brain’s immune environment, leading to different immune responses post-ICH. In the elderly, senescent cells affect the brain’s microenvironment by secreting SASP, including pro-inflammatory signals such as IL-1α, IL-1β, and IL-6. These pro-inflammatory factors affect the function of brain cells [92], with microglia playing a crucial role in this process. Microglia are not only the protagonists in the inflammatory response after ICH but also in brain immune aging and inflammation. Post-ICH, microglia can phagocytose erythrocytes by transitioning to an M2 phenotype (anti-inflammatory phenotype) to clear hematomas. Simultaneously, microglia can be activated to an M1 phenotype (pro-inflammatory phenotype), inducing neuroinflammation and leading to brain edema after ICH. Normally, the homeostatic state of microglia is maintained by the interaction between ligands expressed by neurons (such as CD200, CXCL1, and CD47) and receptors on microglia [93]. However, with aging, this balance is disrupted, tilting the microglial transcriptomic profile towards a chronic inflammatory state, leading to the upregulation of cytokine production, host defense, and cell adhesion genes [94,95,96]. Simultaneously, in aging microglia, levels of TNFα, IL-1β, and IL-6 are elevated, while anti-inflammatory cytokines such as IL-10 are reduced, indicating their diminished capacity to suppress and regulate pro-inflammatory microglial pathways [97]. The accumulation of Aβ during the normal aging process also leads to increased levels of pro-inflammatory cytokines in microglia [93,98]. Studies have found that in the aging brain, there are dysfunctional subpopulations of microglia exhibiting a specific phenotype characterized by increased oxidative stress, enhanced inflammatory response, abnormal lipid accumulation, and increased lysosomal storage [99,100]. Microglia also activate cerebral endothelial cells through the release of TNF-α, specifically upregulate adhesion molecules (VCAM1 and ICAM1) to promote the transendothelial migration of T cells, and release CCL3 to recruit peripheral T cells to the aging brain [101]. These findings imply that with aging, microglia transition into a pro-inflammatory state, thereby contributing to chronic damage in brain tissue, leading to cerebrovascular damage and promoting the occurrence of ICH, but this also results in more severe inflammatory responses in elderly patients after ICH, making recovery more difficult.

As microglia age, their transcriptome also changes, characterized by enhanced inflammation, impaired phagocytic function, and morphological alterations, reducing their immune surveillance capabilities [102]. Animal studies have observed that in aged mice, numerous phagocytic receptors on microglia are downregulated, such as a decrease in TREM2 (a receptor for Aβ phagocytosis) protein expression, suggesting an impairment in the phagocytic function of aged microglia [103]. The diminished clearance capacity of aged microglia leads to slow hematoma absorption after ICH, thereby prolonging the duration of blood toxicity damage to the brain.

An animal study found that in aged mice, the use of microglia replacement therapy can reduce neurofunctional impairments and cerebral edema subsequent to ICH. Additionally, microglia replacement can inhibit the infiltration of leukocytes into the brain and the inflammatory activity of brain microglia, as well as decrease neuronal death after ICH. This suggests that microglia replacement therapy may be a promising candidate in preclinical studies for ICH [104].

Before or after the occurrence of ICH in the elderly, damaged cerebral blood vessels or brain tissue cells release danger-associated molecular patterns (DAMPs), which stimulate inflammatory responses and interact with the pattern recognition receptors on peripheral immune cells. This interaction triggers an inflammatory response, including the release of cytokines [105]. Meanwhile, due to the loss of BBB integrity, aging peripheral immune cells (such as neutrophils, dendritic cells, and natural killer cells) opportunistically invade the brain in large numbers, further exacerbating CNS damage. Peripheral immune cells rapidly recognize and respond to DAMPs in a non-specific manner [106,107]. Their activation and infiltration intensify the secondary brain damage after ICH, leading to more difficult recovery in elderly ICH patients.

Macrophages are the ‘scavengers’ of the immune system, clearing cell debris and pathogens through phagocytosis. As we age, macrophages infiltrate the brain. When these macrophages are exposed to the aging brain environment, they show an increased expression of markers such as MHCII and CD40, producing higher levels of inflammatory mediators, which adversely affect the function of neurons and synapses [108]. For instance, in the brains of mice equivalent to middle-aged humans, the levels of infiltrating macrophages and pro-inflammatory cytokines were higher than in mice that were six months old [109]. An increase in pro-inflammatory phenotypes of macrophages in the brain was found in accelerated aging mouse models [110], indicating that aging promotes the transition of macrophages from blood to the brain, becoming pro-inflammatory cells. 

Neutrophils, akin to macrophages and microglia, play a pivotal role in the anti-inflammatory immune response through their capacity to phagocytose cellular debris. Aging neutrophils exhibit functional impairments in clearing debris and producing enzymes necessary for vascular remodeling. The declined chemotaxis and accuracy in migration towards inflammatory stimuli of aging neutrophils result in defective recruitment [111]. These functional changes in aging neutrophils not only exacerbate chronic inflammation in healthy tissues but also affect recovery from ICH. The functionality of aged dendritic cells is also affected; this includes a diminished capacity to stimulate T-cell proliferation and IFN-γ secretion, diminished efficacy in antigen processing, and decreased clearance of apoptotic cells, leading to prolonged exposure to self-antigens and promoting chronic autoimmune inflammation [112,113]. Astrocytes expressing IL-15 enhance the differentiation of microglia towards a pro-inflammatory phenotype, worsening the neurological prognosis [114].

In conclusion, the aging process not only compromises the integrity of the BBB but also alters the functionality of immune cells both within and external to the brain’s milieu. These aging immune cells lead to chronic, sustained inflammation prior to the occurrence of ICH, resulting in further damage to brain tissue, brain function, and cerebrovascular health. Moreover, the substantial influx of these cells into the brain after ICH exacerbates the post-ICH inflammatory response, thereby negatively impacting the recovery and prognosis of elderly patients with ICH.

### 3.4. ICH Accelerates Aging

#### 3.4.1. Physical Aging

The long-term survival rate of ICH patients is not ideal. For those who survive more than 30 days after ICH onset, less than half of the patients survive for one year, and less than one-third survive for five years [115]. A prospective cohort study involving 304 patients who survived more than 30 days after ICH found that the 10-year cumulative survival rate of ICH patients was only 38% [116]. Survival rates are associated with age, previous comorbidities, clinical severity at presentation (higher NIHSS scores and worse mRS scores), and significant clinical events occurring after ICH. In a single-center study, researchers used the mRS score to evaluate patients who survived six months after ICH, finding that nearly a quarter of ICH patients experienced functional decline over time [117]. This suggests that patients post-ICH may have long-term deterioration in physical function, possibly rendering them unable to independently perform daily living activities. Moreover, multiple studies indicate that only 14–36% of ICH patients achieve functional independence within one year [115,118,119].

Losing independence in daily living and motor abilities not only increases the risk of muscle atrophy and fractures but also elevates the likelihood of cardiovascular diseases. A study revealed differences in adverse cardiovascular events between ICH and IS survivors. Compared to IS survivors, adverse cardiovascular events were more common in ICH survivors, with newly developed heart failure being a relatively frequent occurrence in patients with concomitant atrial fibrillation (AF) after ICH [120]. Another study found that ICH survivors had a high risk of major cerebral and extracerebral ischemic or hemorrhagic events within 5 years. Among the 310 ICH patients included in the study, 82 experienced at least one major vascular event within five years, with a stronger ischemic risk for ICH occurring in deep locations [121]. Regarding the occurrence of IS after ICH, a population-based large-cohort study showed a significant rise in IS risk during the initial 6 months after diagnosing ICH in elderly patients [122]. As for the recurrence of ICH after the initial event, a meta-analysis focusing on ICH research suggested that the annual risk of recurrence within the first year post-ICH ranged between 1.8% and 7.4%, with the annual recurrence risk during the entire study period remaining between 2% and 2.4% [115]. The recurrence risk differed between LICH and non-LICH, with LICH patients having more than double the risk of recurrence compared to non-LICH patients, approximately 7.8% [123]. Research indicates that the likelihood of recurrent ICH, IS, and other major vascular incidents post-ICH is influenced by the hemorrhage’s site and the concurrent presence or absence of AF. Notably, in cases of LICH without accompanying AF, the probability of a subsequent ICH surpasses that of an IS [124].

The decline in bodily functions and the risk of recurrent adverse cardiovascular diseases make rehabilitation more challenging for ICH patients. Researchers from Japan and Singapore investigated the functional outcomes and Return to Work (RTW) capabilities of post-stroke survivors. They consistently found that ICH patients had more difficulty in returning to work compared to IS patients [125,126]. Recovery from ICH takes months, and the decline in bodily functions often prevents returning to work. Prolonged cognitive challenges and reduced social interactions, along with the loss of labor income and social life, can lead to damage to the patients’ self-identity and self-worth, affecting their mental and physical health. Additionally, the recurrence of adverse cardiovascular events and more challenging rehabilitation make it harder for ICH patients to independently perform daily life activities, impacting recovery in various aspects. For example, difficulty eating independently can lead to insufficient or imbalanced nutrition intake, affecting recovery. Lack of activity can decrease sleep quality and increase the risk of diabetes, obesity, and other chronic diseases. These factors can put the brain and body in a state of ‘abandonment’, accelerating psychological and physiological aging.

#### 3.4.2. Mental Aging

Post-stroke cognitive impairment and dementia (PSCID) refer to CI and dementia caused by stroke, which are major sources of morbidity and mortality worldwide after stroke [127]. Most studies investigating CI after ICH focus on the presence of dementia, commonly using the Mini-Mental State Examination (MMSE) or Montreal Cognitive Assessment (MoCA). Yu et al. analyzed MMSE scores in 231 ICH survivors and found that 75 (32.5%) had CI, and the risk of CI increased by 2.48 times for every 10-year increase in age [128]. After a stroke, the risk of dementia following ICH is significantly higher than that after IS. Pendlebury et al. found in the population-based Oxford Vascular Study (OXVASC) that the risk of dementia after ICH is 4.5 times higher than that after IS, while the risk of dementia after IS is 2.5 times higher than normal [129]. A study found that among 208 patients with acute ICH, nearly half showed significant CI during the acute phase of ICH (within one week of hospitalization) [130]. However, CI is not limited to the acute phase; cognitive function in ICH patients continues to decline over time. Long-term follow-up for more than one year after ICH indicates a gradual increase in CI [131]. The increased risk of delayed CI is independently associated with lower MoCA scores during the acute phase [130].

Banerjee et al. conducted neuropsychological assessments on 187 ICH patients across seven cognitive domains (premorbid intellectual functioning, current intellectual functioning, verbal and visual memory, naming, visuospatial perception, information processing speed, and executive function) and found that deficits in non-verbal IQ (76.6%), information processing speed (62.4%), and executive function (58.1%) were the most common. In total, 84% of patients had impairments in at least one cognitive domain, and 65% had impairments in two or more domains [132]. In this study, patients with LICH had more impairments in naming and visuospatial perception, while patients with deep hemorrhage had higher rates of executive function and information processing speed impairments. Additionally, patients with CAA appeared to have distinct cognitive characteristics, with more significant deficits in verbal IQ and executive function among ICH patients with CAA [132].

Not only CI and dementia but also anxiety and depression can cause long-term harm to the physical and mental health of ICH patients. A survey of ICH patients who survived more than one year found that 17% of the patients experienced anxiety within 1–2 years, 27% showed signs of anxiety 3–5 years later, and 21% still exhibited anxiety symptoms 6–8 years later. Additionally, more than half of these anxious patients also exhibited symptoms of depression, with a significant correlation found between LICH and anxiety during the first 1–2 years post-ICH [133]. Studies on post-ICH depression discovered that compared to patients with hypertension-related ICH, survivors of CAA-related ICH were more prone to depression both before and after the onset of ICH, and their depressive symptoms were more likely to persist and show resistance to antidepressant treatments [134]. Previous research on ICH may have focused more on individual reasons affecting ICH, but now, there is increasing evidence that the prevention, treatment, and prognosis of ICH are closely related to social and environmental factors. A study introduced the concept of ‘accelerated aging’ as a physiological age exceeding the actual age. Utilizing data from the Coronary Artery Risk Development in Young Adults (CARDIA) study, it was found that the physiological age of 2978 participants was 3 years older than their actual age, and this physiological age was associated with certain social factors (such as depression and social participation), suggesting that social and environmental factors may accelerate aging in patients with cardiovascular diseases [135].

CI, dementia, anxiety, and depression that occur after ICH can lead to social isolation, resulting in compromised mental health and difficulty in maintaining conversations or social interactions. This decreases social participation among ICH patients, leading them to feel unwanted or ‘abandoned’ by society. Moreover, CI and emotional disorders can reduce compliance, becoming obstacles to behavioral changes and adopting healthy lifestyles, impacting the patient’s rehabilitation treatment and quality of life, and potentially leading to accelerated aging. Conversely, social isolation and loneliness can exacerbate CI and emotional disorders, further accelerating aging. Therefore, in clinical practice, it is vitally important to identify high-risk patient subgroups who develop CI, anxiety, and depression after ICH, in order to provide them with more psychological and social support and interventions, thereby facilitating their better recovery. We also noted that in ICH patients, LICH exhibits different characteristics in the recurrence of ICH, CI, dementia, and anxiety compared to non-LICH, which may be related to the fact that CAA-associated ICH occurs more often in the lobes. This not only warrants deeper research into this area but also suggests that targeted diagnosis and treatment should be provided for patients with LICH and non-LICH.

## 4. Conclusions

Aging is a complex physiological process with multifaceted interactions with ICH. By exploring the mechanistic links between ICH and aging, we have uncovered unique attributes in elderly individuals concerning susceptibility and adverse outcomes following ICH. The pathogenesis of ICH involves multiple factors, including alterations in vascular structure and function, heightened inflammatory responses, and compromised immune function, all closely intertwined with aging. At the same time, we cannot overlook the impact of ICH on cognitive abilities, quality of life, and lifespan. Providing targeted diagnosis and treatment for elderly patients with ICH, who exhibit diverse symptoms, is crucial for effectively reducing the harm ICH causes in the elderly. As the world’s population increasingly ages, it becomes imperative to pay adequate attention and exercise caution regarding the dangers posed by ICH. Consequently, it is essential and should be a continuous effort to intensify research on ICH among the elderly population.
